# Mechanisms of Transmission and Adaptation of *tet*(X4)-Positive IncHI1 Plasmids in XDR *Escherichia coli* from Pet Dogs: The Role of *trhC*, *rsp*, and the Tra1 Region

**DOI:** 10.3390/vetsci12050418

**Published:** 2025-04-28

**Authors:** Pengyun Ding, Qianqian Wang, Liangliang Wang, Mengxiang Zheng, Yiming Feng, Yakun Xu, Li Yuan, Gongzheng Hu, Yushan Pan, Dandan He

**Affiliations:** 1 College of Veterinary Medicine, Henan Agricultural University, Zhengzhou 450046, China; dpy980408@163.com (P.D.); 15225074082@163.com (Q.W.); 2021207066@stu.njau.edu.cn (L.W.); 15138379453@163.com (M.Z.); fym138351@163.com (Y.F.); yuanli-hn@163.com (L.Y.); yaolilab@126.com (G.H.); 2 Ministry of Education Key Laboratory for Animal Pathogens and Biosafety, Zhengzhou 450046, China

**Keywords:** IncHI1 plasmid, *tet*(X4), MDR, conjugative helper plasmid, IS*Cro1*, fusion plasmid

## Abstract

*tet*(X4)-positive IncHI1 plasmids are widely prevalent in various bacteria. To understand their transmission characteristics, we analyzed two extensively drug-resistant (XDR) *Escherichia coli* strains isolated from pet dog feces in Henan Province, China. Strain T28R harbored *tet*(X4)-positive IncHI1, IncF18:A-:B-, and *mcr-1*-positive IncI2 plasmids, while T16R carried *tet*(X4)-positive IncHI1, F16:A-:B-, and *mcr-1*-positive IncX4 plasmids. Four representative fusion plasmids, pT28R-F1, pT28R-F2, pT28R-F3, and pT16R-F1, in transconjugants were analyzed using WGS and PCR mapping. The results showed that IS*26* from the IncF18:A-:B--plasmid attacked the conjugative transfer-associated genes *trhc* and *rsp* on the IncHI1 plasmid, generating pT28R-F1 and pT28R-F2. pT28R-F3 was generated through IS*Cro1*- and IS*CR2*-mediated homologous recombination, deleting the Tra1 region of the IncHI1 plasmid. T16R-F1 emerged from IS*CR2*- and IS*1B*-mediated homologous recombination, losing transfer regions of parental plasmids. Notably, fusion plasmids lost the temperature sensitivity of the IncHI1 plasmid, with conjugation frequencies between 1.57 × 10^−4^ and 3.84 × 10^−5^ at 28 °C and 37 °C. The findings suggest that *tet*(X4)-positive IncHI1 plasmids could be mobilized with the assistance of conjugative helper plasmids and that fusion events enhance the adaptability of these plasmids, thus facilitating the spread of antibiotic resistance, posing a growing public health threat.

## Simple Summary

Tigecycline is one of the last-resort drugs to treat serious infections caused by MDR Gram-negative bacteria. However, the emergence of the plasmid-mediated high-level tigecycline resistance gene *tet*(X4) is bound to create difficulties for clinical treatment. The IncHI1 plasmid serves as a crucial carrier for the transfer of the *tet*(X4) gene, playing a crucial role in the spread of tigecycline resistance. Nevertheless, the conjugative transfer of IncHI1 plasmids exhibits temperature sensitivity. In our study, we discovered that two novel types of IncFII plasmids can act as conjugative helper plasmids to fuse with IncHI1 plasmids through different mechanisms, eliminating temperature sensitivity and promoting their dissemination. Our findings provide new insights into the evolution and transmission of *tet*(X4)-positive IncHI1 plasmids, which are of significant importance for controlling the spread of multidrug-resistant plasmids among organisms.

## 1. Introduction

Tigecycline is one of the last-resort antibiotics used to treat complicated infections caused by both multidrug-resistant Gram-negative and Gram-positive bacteria [1]. However, the recent emergence and spread of novel tigecycline resistance mechanisms, including *tet*(X3), *tet*(X4), *tet*(X5), *tet*(X6), and other variants, have compromised its clinical efficacy [2,3]. Among these *tet*(X) variants, *tet*(X4) has recently been detected in food-producing animals and is the most prevalent *tet*(X) subtype in China [4,5,6]. Plasmids play an important role in the accumulation and transmission of *tet*(X4) genes, and the IncHI1 plasmid is one of the crucial carriers of the *tet*(X4) gene [7,8,9].

Conjugative plasmids are plasmids that facilitate horizontal gene transfer through bacterial conjugation. The dissemination of plasmids promotes genetic diversity and evolution in bacteria, ultimately leading to the spread of antibiotic resistance and pathogenicity [10,11,12]. Notably, recent studies have increasingly highlighted the role of conjugative plasmids in facilitating the mobilization and transfer of clinically important resistance genes and virulence-associated plasmids. In this regard, Chen et al. [13] reported the fusion of non-conjugative PMQR-carrying IncX1 plasmids with a conjugative helper Incl1 plasmid, facilitating the horizontal transmission of ciprofloxacin resistance. He et al. [14] reported the fusion of a conjugative *bla*_CTX-M-55_-carrying IncF33:A-B-plasmid with a non-conjugative, *mcr-1*-carrying phage-like plasmid, promoting the horizontal transfer of *mcr-1*. Wang et al. [15] reported the fusion of the conjugative helper plasmid IncN3 with a non-conjugative pLVPK-like virulence plasmid, expanding the dissemination range of virulence plasmids. Insertion sequences (such as IS*26*, IS*1216E*, and IS*CR2*) and transposons (such as Tn*6952*, Tn*pA21*, and *Tnp*U1548) mediate intermolecular replicative transposition and homologous recombination via homologous regions that play an important role in plasmid fusion events [16,17,18,19,20]. The fusion of plasmids not only expands the host range but also increases the number of antibiotic resistance and virulence genes carried, enabling the fused plasmids to be co-selected under various drug pressures, posing a significant threat to public health.

The prototypic IncHI1 plasmid R27, initially discovered in *Salmonella*, has been extensively studied due to its large molecular weight and temperature-dependent conjugation ability [21,22]. The optimal conjugation rate of IncHI1 plasmids is typically within the range of 22–28 °C, while their conjugation is inhibited at 37 °C and is almost undetectable [23]. Previous studies have indicated that the expression of *trhR*, *trhY*, and *traG* in the Tra1 region, as well as *htdA* in the Tra2 region, is closely associated with temperature-dependent conjugation [21,24]. In this work, we find that *tet*(X4)-positive IncHI1 plasmids in two XDR *E. coli* strains display a relatively high ability of conjugative transfer at 37 °C. Here, we analyze the characteristics of two XDR *E. coli* strains isolated from pet dogs and elucidate the mechanism of fusion between *tet*(X4)-positive IncHI1 plasmids and conjugative helper plasmids, as well as the biological properties of the fused plasmids.

## 2. Materials and Methods

### 2.1. Bacterial Strain

In 2019, fecal samples were collected from pet cats and dogs at veterinary hospitals in Henan Province, China. The strains were incubated on McConkey AGAR plates for 16 to 18 h at 37 °C, purified several times, and then the strain type was confirmed using the VITEK-2 Compact system (Biomérieux, Marcy l’Etoile, France) and *16S rRNA* gene sequencing.

### 2.2. Antimicrobial Susceptibility Testing

Based on the CLSI standards [25], broth microdilution was employed to determine the MIC values of 13 antimicrobials, including tigecycline, eravacycline, omadacycline, tetracycline, doxycycline, ampicillin, cefotaxime, colistin, florfenicol, amikacin, kanamycin, ciprofloxacin, and imipenem. The results were interpreted by matching the breakpoints of the CLSI standards. For tigecycline and florfenicol, the resistant breakpoint was interpreted according to EUCAST (http://www.eucast.org; accessed on 1 January 2024). *E. coli* ATCC 25922 served as the quality control strain.

### 2.3. Conjugation and S1-PFGE

The self-transferability of plasmids from strains T28R and T16R of *E. coli* was assessed using *E. coli* C600 (resistant to rifampicin [rif^r^]) as the recipient. The donors and recipients were inoculated in a broth test tube, and 1 mL of donor bacteria solution was added to 4 mL of recipient bacteria solution after incubation at 130 rpm 37 °C for 4 h. After incubation at 37 °C for 5 h, 2 mL of the mixed bacterial solution were centrifuged, and transconjugants were screened on MacConkey agar plates supplemented with various combinations of dual antibiotics: rifampicin (300 mg/L) paired with either colistin (2 mg/L), tigecycline (2 mg/L), or florfenicol (20 mg/L). Drug resistance genes in the transconjugants were identified through PCR screening using corresponding primers detailed in Table S1. The plasmid profiles of the parental strains and transconjugants were verified using S1-PFGE. Under conditions of 6 V/cm, 14 °C, and a pulse time of 2.16 to 63.8 s for 18 h, plasmid DNA was linearized using S1 nuclease and separated by pulsed-field gel electrophoresis (PFGE) using a CHEF-Mapper system. After electrophoresis, the gel was stained with ethidium bromide and plasmid sizes were estimated by comparison with molecular weight markers. Interestingly, the plasmid profiles identified in the donor and transconjugant strains through S1-PFGE showed that the plasmids in the *tet*(X4)-positive transconjugants C600-pT28R-F1/F2/F3 and C600-pT16R-F1 are different from all the plasmids found in the parental strains T28R or T16R (Figure S1).

### 2.4. WGS and Analysis

To investigate the genetic basis of plasmid size alterations in both donor and transconjugant strains, the total genomic DNA was individually extracted from strains T28R, T16R, and their corresponding transconjugants C600-pT28R-F1/F2/F3 and C600-pT16R-F1 using the QIAamp DNA Mini Kit (Qiagen, Hilden, Germany). Subsequently, WGS was performed using Illumina Nextseq 500 and Oxford Nanopore Technologies (ONT) MinION platforms. The sequencing reads encompassing short-read and long-read data were assembled using unicycler 0.4.4 with a hybrid strategy [26,27]. The plasmid sequences were first annotated using the RAST server (http://rast.nmpdr.org; accessed on 13 January 2024) and then manually corrected. Antimicrobial resistance genes (ARGs), plasmid replicon types, and serotypes were identified using ResFinder (version 4.6), PlasmidFinder (version 2.1), and SerotypeFinder (version 2.0) on the CGE server (https://www.genomicepidemiology.org/). IS elements were determined using ISfinder (https://isfinder.biotoul.fr/; accessed on 1 February 2024). The comparative analysis and generation of plasmid maps were carried out using Easyfig and BRIG [28,29].

### 2.5. Conjugation Frequencies

To determine the conjugative transferability of fused plasmids, conjugation experiments were conducted using C600 strains carrying the parental plasmids pT28R-1 and pT28R-2 and the fused plasmids pT28RF1/F2/F3 as the donor strains, respectively, with J53 as the recipient strain. The donors and recipients were inoculated in a broth test tube, and 1 mL of donor bacteria solution was added to 4 mL of recipient bacteria solution after incubation at 130 rpm 37 °C for 4 h. After incubation at 37 °C for 5 h, 2 mL of the mixed bacterial solution were centrifuged, and the centrifuged bacterial solution was spread on MacConkey AGAR plates containing 2 µg/mL tigecycline and 150 µg/mL sodium azide. After incubation at 37 °C for 16–18 h, the presence of the *tet*(X4) gene and fusion sites in the transconjugant was further confirmed by PCR screening with specific primers. Since IncHI1 is a temperature-sensitive plasmid, we assessed whether this property persisted after co-integration by performing the conjugation frequency test at both 28 °C and 37 °C (Table 1).

### 2.6. Plasmid Stability

The transconjugants were passaged in LB broth without antibiotics for 14 days with continuous 1 × PBS dilution of the culture, followed by plating onto LB agar without antibiotics. Multiple clones were then transferred to LB agar plates supplemented with 2 mg/L of tigecycline. This experiment was conducted in triplicate. All clones grown on agar supplemented with antibiotics were confirmed for the presence of *tet*(X4), *floR*, *fosA3*, and fusion sites using PCR with primers including *tet*(X4)-F/R, *floR*-F/R, and *fosA3*-F/R, as well as specific primer pairs for amplifying regions spanning the fusion junctions (Table S1).

### 2.7. Nucleotide Sequence Accession Numbers

The WGS data of strains T16R and T28R can be accessed through the BioProject IDs PRJNA594788 and PRJNA609210, respectively. Four fusion plasmids were submitted to GenBank with the following accession numbers: pT28R-F1(PP215914), pT28R-F2(PP215915), pT28R-F3(PP215916), and pT16R-F1(PP215913).

## 3. Results

### 3.1. Characterization of tet(X4)-Positive E. coli Strains T28R and T16R

Two tigecycline-resistant *E. coli* strains, namely T16R and T28R, were recovered from fecal samples of healthy pet dogs at a pet hospital in Henan Province, China, in August 2019. Antimicrobial susceptibility testing showed that the XDR strains were resistant to ampicillin, cefotaxime, tetracycline, doxycycline, tigecycline (MIC = 32 mg/L), colistin, fosfomycin, florfenicol, ciprofloxacin, amikacin, and kanamycin, but susceptible to imipenem (Table S2). The strains also exhibited high MICs for the FDA’s newly approved eravacycline (8 mg/L) and omadacycline (16 mg/L). PCR and sequencing confirmed the presence of both the *tet*(X4) gene and *mcr-1*. Sequence analysis revealed that T28R carried 18 antimicrobial resistance genes and belonged to H30:O184-ST7366, whereas T16R carried 20 antimicrobial resistance genes along with manganese/mercury metal resistance clusters and belonged to H20:O159-ST48, associated with the dissemination of Tet(X4), MCR, and NDM [30,31,32,33]. Strikingly, both strains harbored virulence genes *gad* (glutamate decarboxylase), *cma* (colicin M), *iroN* (enterobactin siderophore receptor protein), and *iss* (increased serum survival) (Table S3).

### 3.2. Analysis of Plasmids in T28R and T16R

*tet*(X4) was located on a 190,391 bp plasmid pT16R-1 in T16R and on a 193,098 bp plasmid pT28R-1 in T28R. Both plasmids belonged to the IncHI1 group containing RepHI1A, RepHI1B, and RepFIA replicons and harbored two separated transfer regions, Tra1 and Tra2 (Figure S2A). They carried six resistance genes, including *qnrS1*, *aadA22*, *lnu*(G), *floR*, *bla*_TEM-1B_, and *tet*(A), conferring resistance to quinolone, aminoglycoside, lincosamide, phenicol, β-lactam, and tetracycline, respectively. Sequence analysis revealed a 2699 bp IS*Cro1* mobile element inserted into the target site (TAACCTTT) in pT16R-1, resulting in the formation of pT28R-1 and generating an 8-bp target site duplication (TSD) (TAACCTTT) in pT28R-1 (Figure S2A).

Compared with R27 (AF250878), the prototype of IncHI1 plasmids in *Salmonella typhi*, pT16R-1 and pT28R-1 obtained an MRR and an IS*Pst2*-flanked transposon carrying macrolide resistance gene *Inu*(G), but lacked Tn*10* carrying the *tet*(B) determinant (Figure S2A). The MRR, bounded by two IS*1* copies in the same orientation, can be divided into five structural units. The first segment, IS*1*-*aadA22*-*intI1*-IS*26*, was similar to that of the pZYST1C2 (CP031615) in a clinical *K. pneumoniae* isolate from swine. The other four segments containing IS*26*-∆Tn*3*-*bla*_TEM-1_-*tnp*-IS*26*, IS*26*-*qnrS1*-IS*26*-∆Tn*2*, *lysR*-*floR*-*virD2*-IS*CR2*, and ∆IS*CR2*-*hp*-IS*CR2*-*tet*(X4)-*abh*-IS*1*, showed high similarity with the corresponding structures in the p47EC in an *E. coli* isolate from swine, but their arrangements were different (Figure S2B).

The *mcr-1*-bearing pT16R-3 (33,309 bp) from T16R did not carry any additional known antimicrobial resistance gene and exhibited high similarity (99–100% identity at 100% coverage) with the MCR-1-producing IncX4 plasmids in GenBank (Figure S3a). The *mcr-1*-bearing pT28R-3 (65,023 bp) from T28R belonging to the IncI2 plasmid carried the *bla*_CTX-M-64_ gene and shared the highly conserved IncI2 backbone of *mcr-1*-bearing plasmids (Figure S3b). A copy of IS*2* was inserted into pT28R-3, bringing the appearance of a 5-bp TSD (AATGA), and was absent in other *mcr-1*-positve IncI2 plasmids (Figure S3b).

The *fosA3*-carrying chromosome in *E. coli* T16R harbored other resistance genes, including *bla*_CTX-M-14_, *rmtB*, *bla*_TEM-1_, and *mdf*(A). A 10,944 bp MRR bracketed by two intact IS*26* was located on an intact prophage region on a chromosome that was identified using PHASTER (http://phaster.ca/; accessed on 1 February 2024), and contained three IS*26*-flanked transposon IS*26*-*fosA3*-1649bp-IS*26*, IS*26*-*rmtB*-*bla*_TEM-1_-IS*26*, and IS*26*-ΔIS*Ecp1-bla*_CTX-M-55_-∆*bla*_TEM-1_-IS*26* that had repeatedly been located on different plasmids or chromosomes of different bacteria [34,35]. Importantly, the tandem arrays of three IS*26*-associated modules emerged for the first time in the prophage region of the chromosome (Figure S4). In *E. coli* T28R, the *fosA3* gene was identified in a 161,057 bp IncF18:A-:B-plasmid pT28R-2, along with other resistance genes containing *bla*_CTX-M-14_, *bla*_TEM-1_, *floR*, *dfrA12*, *mph*(A), *sul2*, *aadA2*, *aac(3)-IV*, *aph(3′)-Ia*, and *aph(4)-Ia*. In addition, a 172,892 bp IncF16:A-:B1 plasmid pT16R-2 in T16R carried manganese/mercury resistance clusters (*merT/P/C/E* and *sitA/B/C/D*) and antibiotic resistance genes, including *bla*_TEM-1_, *tet*(A), *strB/A*, *floR*, *aph(3′)-Ia*, *mph*(A), *sul1/2*, *aadA5*, and *drfA17* (Table S3).

### 3.3. Multimodal Transmission of tet(X4) in a IncHI1 Plasmid Mediated by IS Elements

An S1-PFGE analysis indicated that in the transconjugants of strain T28R, the *tet*(X4)-positive plasmids (ranging from 260 kb to 350 kb) were larger than any of the original plasmids detected in the donor strain T28R. We selected three representative plasmids for further analysis. Based on their size and antimicrobial resistance profiles, these plasmids were likely generated through recombination events between pT28R-1 (~190 kb) and pT28R-2 (~160 kb). In the transconjugants of strain T16R, several *tet*(X4)-positive plasmids (ranging from 110 kb to 360 kb) were detected that differed from any of the original plasmids in T16R. These were likely formed through recombination between pT16R-1 (~190 kb) and pT16R-2 (~170 kb). As strains T28R and T16R harbor similar plasmids, they may exhibit similar recombination or fusion patterns. Therefore, we focused on analyzing the smaller fusion plasmids from the T16R-derived transconjugant C600-pT16R-F1, as it differed in size from those observed in the T28R-derived transconjugants C600-pT28R-F1/F2/F3.

**Figure 1:**
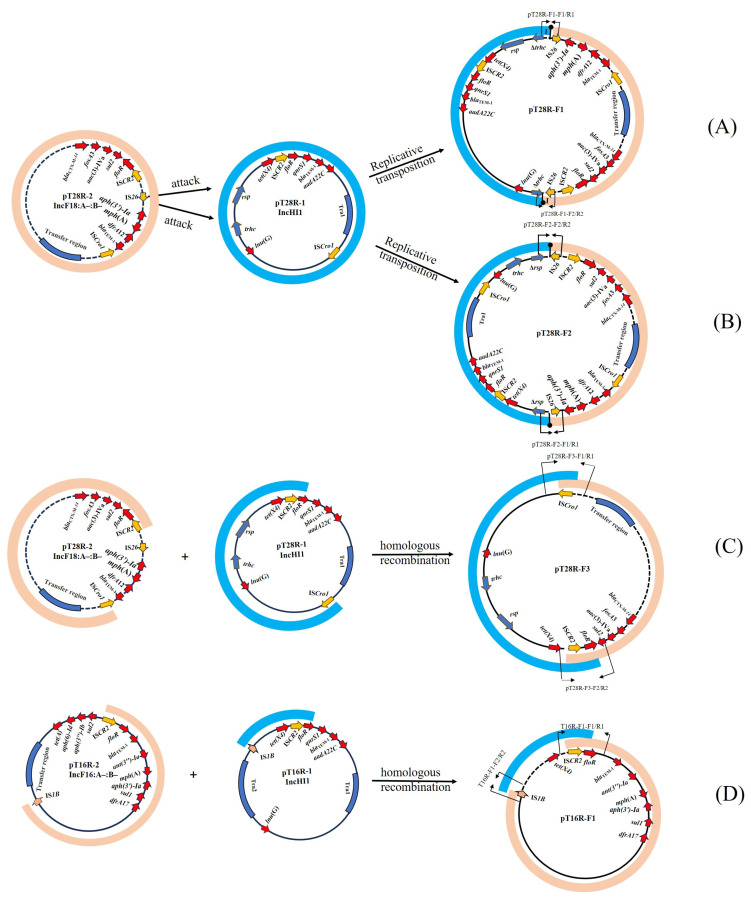
The proposed model for the formation of the fusion of plasmids. (**A**) The fusion plasmid pT28R-F1 was formed via IS*26*-mediated recombination between pT28R-1 and pT28R-2, resulting in the truncation of *trhC* and the creation of an 8-bp target site duplication (GTATTTCC). (**B**) The fusion plasmid pT28R-F2 was formed via IS*26*-mediated recombination between pT28R-1 and pT28R-2, resulting in the truncation of *rsp* and the formation of an 8-bp target site duplication (TTAAATGT). (**C**) The fusion plasmid pT28R-F3 was generated through IS*CR2*- and IS*Cro1*-mediated homologous recombination between pT28R-1 and pT28R-2, leading to plasmid fusion with simultaneous loss of resistance and conjugation-associated regions. (**D**) The fusion plasmid pT16R-F1 was formed via homologous recombination between pT16R-1 and pT16R-2, mediated by IS*CR2* and IS*1B*, resulting in a non-conjugative plasmid retaining the *tet*(X4)-containing fragment and partial resistance regions. The plasmid name is located in the center of each circle. Two pairs of primers crossing the fusion region are marked in black. Yellow arrow: mobile elements; blue arrows: transfer-associated genes;red arrows: resistance genes.Filled circles on stalks with numbers 1 and 2 represent the 8-bp target site (GTATTTCC) and (TTAAATGT) in pT28R-F1 and pT28R-F2, respectively.

**Figure 2:**
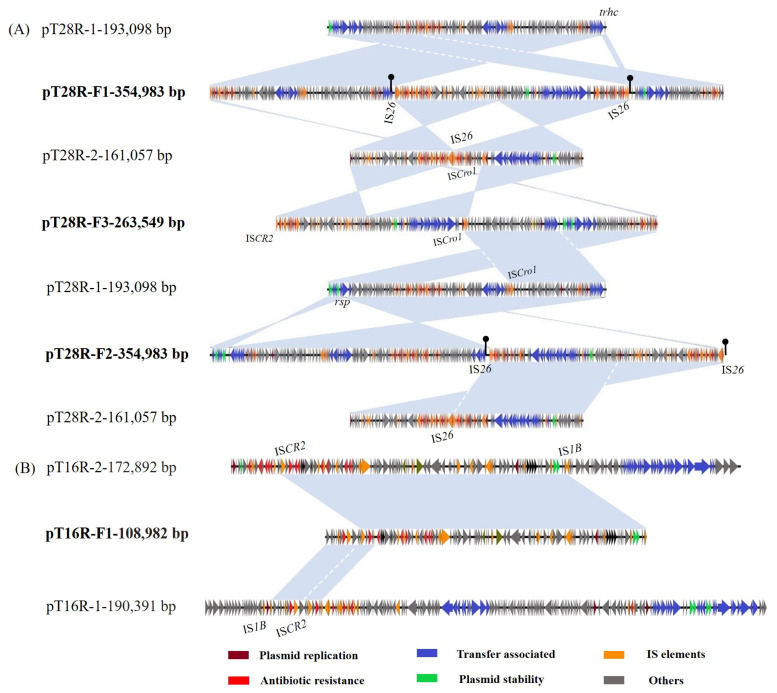
Linear sequence comparison of the parental plasmids and four fused plasmids. (**A**) Linear sequence comparison of parental plasmids pT28R-1 and pT28R-2 with fusion plasmids pT28R-F1, pT28R-F2, and pT28R-F3. (**B**) Linear sequence comparison of parental plasmids pT16R-1 and pT16R-2 with the fusion plasmid pT16R-F1. Colored arrows represent open reading frames, with brown, green, blue, red, orange, and gray arrows representing replicon genes, stability-associated genes, transfer-associated genes, resistance genes, insertion sequences, and hypothetical proteins, respectively. Filled circles on stalks represent the 8 bp target site (GTATTTCC) in pT28R-F1 and the 8 bp target site (TTAAATGT) in pT28R-F3. The shaded area represents 100% identity.

The plasmid pT28R-F1, identified in the conjugant C600-pT28R-F1, was 354,983 bp in length. A proposed model for its formation is shown in Figure 1A. Briefly, the IS*26* element downstream of *floR* on pT28R-2 attacked the target site (GTATTTCC) within the conjugation transfer gene *trhC* in pT28R-1, resulting in the truncation of *trhC*. The linearized pT28R-1 was then incorporated into the pT28R-2, creating the cointegrate pT28R-F1 and creating the appearance of an 8-bp target site duplication (TSD) (GTATTTCC) and the acquisition of an additional copy of IS*26* that was located upstream of the incoming pT28R-2 molecule. Then, the TSDs became two direct repeats surrounding the insertion fragment. pT28R-F1 was a fusion plasmid composed of sequences of pT28R-1 (1–128,086 nt; 295,758–354,983 nt), pT28R-2 (128,085–289,963 nt), and an additional 828-bp sequence (128,079–128,906 nt) containing an IS*26* copy and 8 bp repetitive sequence (GTATTTCC) (Figure 2A).

The plasmid pT28R-F2, identified in the transconjugant C600-pT28R-F2, was 354,983 bp in length. We present a hypothetical model for its formation in Figure 1B, which is similar to the pT28R-F1 model described above, differing only by the target site. Briefly, the IS*26* adjacent to downstream of *floR* in pT28R-2 attacked the target site (TTAAATGT) in the *rsp* (conjugation transfer gene) of pT28R-1, resulting in the truncation of *rsp*. Linearized pT28R-1 was incorporated into the pT28R-2, creating the cointegrate pT28R-F2 and creating the appearance of an 8-bp TSD (TTAAATGT) and the acquisition of an additional copy of IS*26* that was located upstream of the incoming pT28R-2 molecule. Then, the TSD became two direct repeats surrounding the insertion fragment. The resulting plasmid, pT28R-F2, was a fusion plasmid composed of sequences from pT28R-1 (1–193,117 nt), pT28R-2 (193,107–354,983 nt), and an additional 828-bp sequence (193,099–19,926 nt) that included an IS*26* copy and 8 bp repetitive sequence (TTAAATGT) (Figure 2A).

The plasmid pT28R-F3, identified in the transconjugant C600-pT28R-F3, was 263,549 bp in length. A proposed model for its formation is shown in Figure 1C. Sequence analysis indicates that the formation mechanism of this plasmid may be due to the homologous recombination of the 3758 bp homologous region mediated by IS*CR2* and the 2703 bp homologous region mediated by IS*Cro1* in pT28R-1 and pT28R-2. This recombination event resulted in the fusion of the two plasmids and the loss of large fragments, including a portion of the resistance region in pT28R-2 and the Tra1 conjugation transfer region in pT28R-1. The resulting plasmid pT28R-F3 was composed of fragments from pT28R-1 (129,910–263,549 nt) and pT28R-2 (1–132,612 nt) (Figure 2A).

The plasmid pT16R-F1, identified in the transconjugant C600-pT16R-F1, was 108,982 bp in length. A proposed model for its formation is shown in Figure 1D. The formation mechanism of this plasmid may have resulted from homologous recombination involving a 4513 bp homologous region mediated by IS*CR2* and a 769 bp homologous region mediated by IS*1B*. Sequence analysis showed that pT16R-F1 did not acquire any conjugation transfer region sequences from two parental plasmids and only kept part of the backbone and resistance region of pT16R-2, and the *tet*(X4)-containing fragment flanked by IS*CR2* and IS*1B* from pT16R-1. pT16R-F1 was composed of fragments from pT16R-1 (47,460–64,206 nt) and pT16R-2 (12,234–108,982 nt) (Figure 2B).

The sequences spanning the cointegrate junctions were confirmed using primers pT28R-F1-F1/R1 and pT28R-F1-F2/R2 for pT28R-F1, pT28R-F2-F1/R1 and pT28R-F2-F2/R2 for pT28R-F2, pT28R-F3-F1/R1 and pT28R-F3-F2/R2 for pT28R-F3, pT16R-F1-F1/R1 and pT16R-F1-F2/R2 for pT16R-F1, and sequences of PCR amplicons corresponding to the result of WGS. The positions of these primers are indicated in Figure 1. Then, we performed PCR using these primers in the donor strain T28R and 200 transconjugants randomly selected on agar plates containing rifampicin and tigecycline in conjugation using *E. coli* C600 as the recipient strain to check the occurrence frequency of the three fusion plasmids described above. The corresponding PCR products could be generated: 5 (5/200, 2.5%) transconjugants carrying pT28R-F1, 7 (7/200, 3.5%) transconjugants carrying pT28R-F2, and 23 (23/200, 11.5%) transconjugants carrying pT28R-F3. The remaining 165 (165/200, 82.5%) transconjugants may represent other yet unidentified fusion patterns, thus requiring further analysis.

### 3.4. Biological Features of Fusion Plasmids

Fusion plasmids pT28R-F1, pT28R-F2, and pT28R-F3 remained stable (stability around 95%) for 14 days of passage in an antibiotic-free environment, indicating these plasmids had relatively high stability so that the resistance genes in cointegrate can be stably inherited.

Conjugation experiments were conducted to determine the conjugation frequency of the parental plasmids pT28R-1 and pT28R-2 and the fusion plasmids pT28RF1/F2/F3 from *E. coli* C600 to *E. coli* J53. The results show that the conjugation frequency of pT28R-1 at 37 °C decreased by 8000-fold to 2.7 × 10^−7^ compared to 2.28 × 10^−3^ at 28 °C, and pT28R-2 ranged from 1.57 × 10^−4^ to 3.14 × 10^−4^ at both temperatures. The conjugation frequency of pT28R-F1 at 28 °C was 3.65 × 10^−5^, and at 37 °C was 3.84 × 10^−5^. pT28R-F2 exhibited a similar range, between 2.45 × 10^−5^ and 3.62 × 10^−5^. The conjugation frequency of pT28R-F3 was the highest among the three fusion plasmids, ranging from 1.57 × 10^−4^ to 3.14 × 10^−4^ at both temperatures. The conjugation frequency of the three fusion plasmids was stable across different temperatures, indicating that they were temperature-insensitive.

## 4. Discussion

The interactions between plasmids are highly important for their own maintenance and for facilitating conjugative transfer [17,36,37]. In recent years, there has been a growing number of reports on plasmid fusion events, broadening the host range and enhancing the number of antibiotic resistance and virulence genes carried. The fusion between non-conjugative plasmids and conjugative helper plasmids is often associated with events driven by insertion sequences and transposon-mediated intermolecular transposition and homologous recombination within homologous regions [13,38,39,40]. Therefore, plasmids that carry more insertion elements are more likely to serve as helper plasmids, facilitating the transfer of non-conjugative plasmids. Among the reported conjugative helper plasmids, the IncF, Incl, IncN3, IncX1, IncP, and IncF plasmids are relatively common [14,41,42]. IncF plasmids are the predominant plasmid type in *E. coli*, exhibiting a high diversity in backbone size, the number of replication modules, conjugation ability, and often carrying multiple resistance regions (MRRs) mediated by insertion sequence or transposons [43,44,45]. In this study, we discovered for the first time that IncF18:A-:B- and IncF16:A-:B-plasmids can serve as conjugative helper plasmids and undergo fusion with *tet*(X4)-positive IncHI1 plasmids through different mechanisms (Figure S5). This finding further highlights the important role of IncF plasmids in promoting plasmid fusion events.

Among 200 randomly selected transconjugants, the fusion plasmid pT28R-F3 mediated by IS*Cro1* had the highest proportion, followed by pT28R-F2 and pT28R-F1, suggesting that IS*Cro1* is a key contributor to the process of plasmid fusion. This study reported for the first time that the insertion sequence IS*Cro1* of the IS66 family mediates plasmid fusion, which is usually associated with antibiotic resistance genes such as *bla*_CTX-M-15_, *bla*_TEM-1_, *tet* (A), etc., and can promote its transmission in the family *Enterobacteriaceae* [46,47,48]. Surprisingly, the IncHI1 plasmid R27, which was first isolated from *S. enterica* in the UK in 1961, also lacks IS*Cro1*, suggesting that IS*Cro1* may have been acquired by the IncHI1 plasmid of the T28R strain in a later evolutionary event, with supporting evidence from the presence of TSDs flanking IS*Cro1* on the IncHI1 plasmid pT28R-1 from T28R. The presence of IS*Cro1* in the IncHI1 plasmid may contribute to the spread of the plasmid and the acquisition of other antibiotic resistance genes.

The transfer genes of the IncHI1 plasmid are located in two separate regions known as Tra1 and Tra2 [24]. The Tra1 region contains 14 open reading frames (ORFs) encoding proteins for mating pair formation (Mpf) proteins, conjugation, and relaxosome [49]. The Tra2 region is 36 kb in length and has 28 ORFs, 11 of which are essential Mpf genes for conjugative transfer [50,51]. The *trhC* gene, one of the 11 Mpf genes in Tra2, is crucial for both plasmid transfer and H-pilus production. Previous studies have shown that its disruption leads to the loss of conjugative transfer ability in IncHI1 plasmids [52]. The *rsp* gene, also in the Tra2 region, is essential for bacterial flagellar structure and intercellular adhesion, but its inactivation significantly reduces the conjugation frequency, potentially to undetectable levels in experiments [53]. In this study, pT28R-F1 and pT28R-F2 were formed by IS*26* in pT28R-2 attacking *trhC* and *rsp* genes in pT28R-1, respectively, resulting in the truncation of both genes. pT28R-F3 was formed through homologous recombination between T28R-1 and T28R-2, mediated by IS*Cro1* and IS*CR2*, leading to the deletion of the Tra1 region and some antibiotic resistance genes. It is worth noting that the conjugation frequencies of the three fusion plasmids are similar at both 28 °C and 37 °C (1.57 × 10^−4^~2.45 × 10^−5^), indicating that the conjugative transfer of the fusion plasmids is not influenced by temperature. This phenomenon also suggests that the IncHI1 plasmid loses its temperature-sensitive characteristic after fusing with the IncF18:A-:B- plasmid carrying *tet*(X4), thereby promoting its spread under different environmental conditions. On the other hand, this suggests that the *rsp*, *trhC*, and Tra1 regions may be associated with the temperature-sensitive transfer of IncHI1, which requires further experiments to validate this phenomenon.

Generally, an increase in plasmid size and the number of carried genes will result in an increase in the energy and time costs associated with plasmid replication and transfer [54,55]. Our findings in this study support this theory, as the conjugative frequency of the three fusion plasmids was 30–300 times lower than that of the IncF18:A-:B-plasmid (from 1.6 × 10^−2^ to 9.5 × 10^−3^). Moreover, the fusion plasmids had lower conjugation frequencies than the IncHI1 plasmid at its optimal conjugation temperature of 28 °C, with a 20–100 times difference. The conjugation frequency of pT28R-F3 at 28 °C and 37 °C, 1.57 × 10^−4^ and 3.14 × 10^−4^, respectively, was 8–10 times higher than that of pT28R-F1 and pT28R-F2. This difference might be due to the smaller size and fewer resistance genes of pT28R-F3 compared to pT28R-F1 and pT28R-F2. Despite the lower conjugation frequency of fused plasmids than the parental plasmids, they still showed a relatively high conjugative capacity at both temperatures. Furthermore, plasmid stability tests revealed that the three fused plasmids, pT28R-F1, pT28R-F2, and pT28R-F3, maintained a retention rate of approximately 95% after 14 days of passage without antibiotic selection, indicating their high stability within the host bacteria. These findings suggested that IncHI1 plasmids might have evolved to enhance their transmission and stability during the evolutionary process, thereby facilitating the spread of tet(X4) and posing a significant threat to public health.

## 5. Conclusions

In conclusion, the complete nucleotide sequences of the genome and three plasmids in *tet*(X4)-positive XDR *E. coli* strains T28R and T16R isolated from pet dogs were analyzed. The study identified two novel conjugative helper plasmids, IncF18:A-:B- and IncF16:A-:B-, that could be fused with *tet*(X4)-carrying IncHI1 plasmids by IS*26*, IS*Cro1*, and IS*CR2*-mediated modes. This is the first report of plasmid fusion mediated by IS*Cro1*, which is essential for this process. Notably, fusion between IncFII and *tet*(X4)-carrying IncHI1 plasmids showed various patterns and tended to disrupt the conjugative transfer elements of the IncHI1 plasmid, eliminating its temperature-dependent conjugation and promoting its evolution towards easier dissemination and stability. Our findings provide new insights into the evolution and transmission of *tet*(X4)-positive IncHI1 plasmids, which are of significant importance for controlling the spread of multidrug-resistant plasmids among organisms.

## Figures and Tables

**Table 1:** Conjugation frequencies of parental plasmids and fusion plasmids.

Plasmid	28 °C			37 °C		
	Mean	No.	Range	Mean	No.	Range
pT28R-1	2.28 × 10^−3^	3	4.37 × 10^−4^~4.2 × 10^−3^	2.7 × 10^−7^	3	4.4 × 10^−7^~5.2 × 10^−8^
pT28R-2	9.5 × 10^−3^	3	2.23 × 10^−2^~5.15 × 10^−3^	1.6 × 10^−2^	3	1.1 × 10^−2^~4.47 × 10^−3^
pT28R-F1	3.65 × 10^−5^	3	1.2 × 10^−5^~5.35 × 10^−5^	3.84 × 10^−5^	3	2.13 × 10^−5^~5.3 × 10^−5^
pT28R-F2	2.45 × 10^−5^	3	9.2 × 10^−6^~4.32 × 10^−5^	3.62 × 10^−5^	3	4.1 × 10^−6^~5.2 × 10^−5^
pT28R-F3	1.57 × 10^−4^	3	6.23 × 10^−4^~2.7 × 10^−3^	3.14 × 10^−4^	3	2.1 × 10^−4^~7.3 × 10^−4^

## Data Availability

All data are contained in this article.

## Supplementary Materials

The following supporting information can be downloaded at https://www.mdpi.com/article/10.3390/vetsci12050418/s1, Table S1: Primer sequence of this study; Table S2: Antimicrobial susceptibility testing of *E. coli* strains T28R, T16R and the transconjugants in this study; Table S3: Characterization of *E. coli* strains T28R, T16R and their transconjugants used in this study; Figure S1: S1-PFGE of *E. coli* strains T28R, T16R and their transconjugants; Figure S2: Characteristics of the IncHI1 plasmid pT16R-1 and pT28R-1 carrying *tet*(X4); Figure S3: Structural comparisons of *mcr-1*-positive plasmids in this study and the similar plasmids in GenBank; Figure S4: Comparative analysis of the multidrug resistance regions; Figure S5: Comparative analysis of plasmids pT28R-2 and pT16R-2.

## References

[1] He T., Wang R., Liu D., Walsh T.R., Zhang R., Lv Y., Ke Y., Ji Q., Wei R., Liu Z. (2019). Emergence of plasmid-mediated high-level tigecycline resistance genes in animals and humans. Nat. Microbiol..

[2] Li R., Peng K., Li Y., Liu Y., Wang Z. (2020). Exploring *tet*(X)-bearing tigecycline-resistant bacteria of swine farming environments. Sci. Total Environ..

[3] Dai S., Liu D., Han Z., Wang Y., Lu X., Yang M., Zhang Y. (2022). Mobile tigecycline resistance gene *tet*(X4) persists with different animal manure composting treatments and fertilizer receiving soils. Chemosphere.

[4] Sun C., Cui M., Zhang S., Wang H., Song L., Zhang C., Zhao Q., Liu D., Wang Y., Shen J. (2019). Plasmid-mediated tigecycline-resistant gene *tet*(X4) in *Escherichia coli* from food-producing animals, China, 2008–2018. Emerg. Microbes Infect..

[5] Feng J., Su M., Li K., Ma J., Li R., Bai L., Wang X., Wang J., Yang Z. (2022). Extensive spread of *tet*(X4) in multidrug-resistant *Escherichia coli* of animal origin in western China. Veter- Microbiol..

[6] Wu Y., He R., Qin M., Yang Y., Chen J., Feng Y., Liang X., Deng W., Ding X., Qin L.-N. (2022). Identification of plasmid-mediated tigecycline-resistant gene *tet*(X4) in *Enterobacter cloacae* from pigs in China. Microbiol. Spectr..

[7] Ma J., Wang J., Yang H., Su M., Li R., Bai L., Feng J., Huang Y., Yang Z., Tang B. (2023). IncHI1 plasmids mediated the *tet*(X4) gene spread in *Enterobacteriaceae* in porcine. Front. Microbiol..

[8] Zhang Y., Zhang J., Cai P., Lu Y., Sun R.-Y., Cao M.-T., Xu X.-L., Webber M.A., Jiang H.-X. (2023). IncHI1 plasmids are epidemic vectors that mediate transmission of *tet*(X4) in *Escherichia coli* isolated from China. Front. Microbiol..

[9] Yu Y., Cui C.-Y., Kuang X., Chen C., Wang M.-G., Liao X.-P., Sun J., Liu Y.-H. (2021). Prevalence of *tet*(X4) in *Escherichia coli* From Duck Farms in Southeast China. Front. Microbiol..

[10] Couturier A., Virolle C., Goldlust K., Berne-Dedieu A., Reuter A., Nolivos S., Yamaichi Y., Bigot S., Lesterlin C. (2023). Real-time visualisation of the intracellular dynamics of conjugative plasmid transfer. Nat. Commun..

[11] Wang Q., Lei C., Cheng H., Yang X., Huang Z., Chen X., Ju Z., Zhang H., Wang H. (2022). Widespread dissemination of plasmid-mediated tigecycline resistance gene *tet*(X4) in *Enterobacterales* of porcine origin. Microbiol. Spectr..

[12] Allain M., Mahérault A.C., Gachet B., Martinez C., Condamine B., Magnan M., Kempf I., Denamur E., Landraud L. (2023). Dissemination of IncI plasmid encoding *bla*_CTX-M-1_ is not hampered by its fitness cost in the pig’s gut. Antimicrob. Agents Chemother..

[13] Chen K., Dong N., Chan E.W.-C., Chen S. (2019). Transmission of ciprofloxacin resistance in *Salmonella* mediated by a novel type of conjugative helper plasmids. Emerg. Microbes Infect..

[14] He D., Zhu Y., Li R., Pan Y., Liu J., Yuan L., Hu G. (2019). Emergence of a hybrid plasmid derived from IncN1-F33:A−:B− and *mcr-1*-bearing plasmids mediated by IS*26*. J. Antimicrob. Chemother..

[15] Wang X., Tang B., Liu G., Wang M., Sun J., Tan R., Pan T., Qu J., Liu J., Ou H.-Y. (2022). Transmission of nonconjugative virulence or resistance plasmids mediated by a self-transferable IncN3 plasmid from carbapenem-resistant *Klebsiella pneumoniae*. Microbiol. Spectr..

[16] Chen K., Xie M., Chan E.W.-C., Chen S. (2022). Delineation of IS*Ecp1* and IS*26*-mediated plasmid fusion processes by MinION single-molecule long-read sequencing. Front. Microbiol..

[17] Shan X., Yang M., Wang N., Schwarz S., Li D., Du X.-D. (2022). Plasmid fusion and recombination events that occurred during conjugation of *poxtA*-Carrying plasmids in *Enterococci*. Microbiol. Spectr..

[18] Li R., Lu X., Peng K., Liu Y., Xiao X., Wang Z. (2020). Reorganization of *mcr-1*-bearing large MDR plasmids resolved by nanopore sequencing. J. Antimicrob. Chemother..

[19] Liu Y.-Y., He D.-D., Zhang M.-K., Pan Y.-S., Wu H., Yuan L., Liu J.-H., Hu G.-Z. (2021). The formation of two *Hybrid plasmids* mediated by IS*26* and Tn*6952* in *Salmonella enterica* serotype enteritidis. Front. Microbiol..

[20] Gu Y., Lü Z., Cao C., Sheng H., Li W., Cui S., Li R., Lü X., Yang B. (2021). Cunning plasmid fusion mediates antibiotic resistance genes represented by ESBLs encoding genes transfer in foodborne *Salmonella*. Int. J. Food Microbiol..

[21] Gibert M., Juárez A., Zechner E.L., Madrid C., Balsalobre C. (2014). TrhR, TrhY and HtdA, a novel regulatory circuit that modulates conjugation of the IncHI plasmids. Mol. Microbiol..

[22] Taylor D.E., Brose E.C. (1985). Characterization of incompatibility group HI1 plasmids from *Salmonella typhi* by restriction endonuclease digestion and hybridization of DNA probes for Tn*3*, Tn*9*, and Tn*10*. Can. J. Microbiol..

[23] Sherburne C.K., Lawley T.D., Gilmour M.W., Blattner F.R., Burland V., Grotbeck E., Rose D.J., Taylor D.E. (2000). The complete DNA sequence and analysis of R27, a large IncHI plasmid from *Salmonella typhi* that is temperature sensitive for transfer. Nucleic Acids Res..

[24] Gunton J.E., Gilmour M.W., Alonso G., Taylor D.E. (2005). Subcellular localization and functional domains of the coupling protein, TraG, from IncHI1 plasmid R27. Microbiology.

[25] Humphries R., Bobenchik A.M., Hindler J.A., Schuetz A.N. (2021). Overview of changes to the clinical and laboratory standards institute performance standards for antimicrobial susceptibility testing, M100, 31st Edition. J. Clin. Microbiol..

[26] Wick R.R., Judd L.M., Gorrie C.L., Holt K.E. (2017). Unicycler: Resolving bacterial genome assemblies from short and long sequencing reads. PLoS Comput. Biol..

[27] Li R., Xie M., Dong N., Lin D., Yang X., Wong M.H.Y., Chan E.W.-C., Chen S. (2018). Efficient generation of complete sequences of MDR-encoding plasmids by rapid assembly of MinION barcoding sequencing data. GigaScience.

[28] Sullivan M.J., Petty N.K., Beatson S.A. (2011). Easyfigure: A genome comparison visualizer. Bioinformatics.

[29] Alikhan N.-F., Petty N.K., Ben Zakour N.L., Beatson S.A. (2011). BLAST Ring Image Generator (BRIG): Simple prokaryote genome comparisons. BMC Genom..

[30] Chen C., Wu X.-T., He Q., Chen L., Cui C.-Y., Zhang Y., Chen S.-H., Liao X.-P., Liu Y.-H., Sun J. (2019). Complete sequence of a *tet*(X4)-harboring IncX1 plasmid, pYY76-1-2, in *Escherichia coli* from a cattle sample in China. Antimicrob. Agents Chemother..

[31] Wise M.G., Estabrook M.A., Sahm D.F., Stone G.G., Kazmierczak K.M. (2018). Prevalence of *mcr*-type genes among colistin-resistant *Enterobacteriaceae* collected in 2014-2016 as part of the INFORM global surveillance program. PLoS ONE.

[32] Liu Z., Wang Y., Walsh T.R., Liu D., Shen Z., Zhang R., Yin W., Yao H., Li J., Shen J. (2017). Plasmid-mediated novel *bla*_NDM-17_ gene encoding a carbapenemase with enhanced activity in a sequence type 48 *Escherichia coli* strain. Antimicrob. Agents Chemother..

[33] Zhang R., Wang Y., Liu Z., Li J., Yin W., Lei L., Wu C., Shen J. (2015). Characterization of NDM-1-producing carbapenemase in *Acinetobacter spp*. and *E. coli* isolates from diseased pigs. Front. Agric. Sci. Eng..

[34] He D., Liu L., Guo B., Wu S., Chen X., Wang J., Zeng Z., Liu J.-H. (2017). Chromosomal location of the *fosA3* and *bla*_CTX-M_ genes in *Proteus mirabilis* and clonal spread of *Escherichia coli* ST117 carrying *fosA3*-positive IncHI2/ST3 or F2:A-:B- plasmids in a chicken farm. Int. J. Antimicrob. Agents.

[35] Lin D., Chen S. (2015). First detection of conjugative plasmid-borne fosfomycin resistance gene *fosA3* in *Salmonella* isolates of food origin. Antimicrob. Agents Chemother..

[36] He K., Li W., Zhao B., Xu H., Pan Y., He D., Hu G., Wu H., Yuan L. (2022). Spreading Advantages of Coresident Plasmids *bla*_CTX-M_-Bearing IncFII and *mcr-1*-Bearing IncI2 in *Escherichia coli*. Microbiol. Spectr..

[37] Liu Y., Qiao Z., Ma Y., Wang M., Hu G., Li E. (2024). Molecular characterization of the *tet*(M)-carrying transposon Tn*7124* and plasmids in *Escherichia coli* isolates recovered from swine. Front. Veter-Sci..

[38] Yang X., Dong N., Chan E.W.-C., Zhang R., Chen S. (2021). Carbapenem resistance-encoding and virulence-encoding conjugative plasmids in *Klebsiella pneumoniae*. Trends Microbiol..

[39] Heaton M.P., Discotto L.F., Pucci M.J., Handwerger S. (1996). Mobilization of vancomycin resistance by transposon-mediated fusion of a VanA plasmid with an *Enterococcus faecium* sex pheromone-response plasmid. Gene.

[40] Carraro N., Matteau D., Luo P., Rodrigue S., Burrus V. (2014). The master activator of IncA/C conjugative plasmids stimulates genomic islands and multidrug resistance dissemination. PLoS Genet..

[41] Xu Y., Zhang J., Wang M., Liu M., Liu G., Qu H., Liu J., Deng Z., Sun J., Ou H.-Y. (2021). Mobilization of the nonconjugative virulence plasmid from hypervirulent *Klebsiella pneumoniae*. Genome Med..

[42] Zhou Q., Wu C., Zhou P., Zhang J., Xiong Z., Zhou Y., Yu F. (2023). Characterization of hypervirulent and carbapenem-resistant *K. pneumoniae* isolated from neurological patients. Infect. Drug Resist..

[43] Partridge S.R., Zong Z., Iredell J.R. (2011). Recombination in IS*26* and Tn*2* in the evolution of multiresistance regions carrying *bla*_CTX-M-15_ on conjugative IncF plasmids from *Escherichia coli*. Antimicrob. Agents Chemother..

[44] Rodríguez-Martínez J.-M., Lopez-Cerero L., García-Duque A., Rodriguez-Baño J., Pascual A. (2021). Interplay between IncF plasmids and topoisomerase mutations conferring quinolone resistance in the *Escherichia coli* ST131 clone: Stability and resistance evolution. Eur. J. Clin. Microbiol. Infect. Dis..

[45] Pitout J.D.D., Chen L. (2023). The significance of epidemic plasmids in the success of multidrug-resistant drug pandemic extraintestinal pathogenic *Escherichia coli*. Infect. Dis. Ther..

[46] Han C.-G., Shiga Y., Tobe T., Sasakawa C., Ohtsubo E. (2001). Structural and functional characterization of IS*679* and IS*66*-family elements. J. Bacteriol..

[47] Petty N.K., Bulgin R., Crepin V.F., Cerdeño-Tárraga A.M., Schroeder G.N., Quail M.A., Lennard N., Corton C., Barron A., Clark L. (2009). The citrobacter rodentium genome sequence reveals convergent evolution with human pathogenic *Escherichia coli*. J. Bacteriol..

[48] Alvarez-Fraga L., Phan M.-D., Goh K.G.K., Nhu N.T.K., Hancock S.J., Allsopp L.P., Peters K.M., Forde B.M., Roberts L.W., Sullivan M.J. (2022). Differential Afa/Dr fimbriae expression in the multidrug-resistant *Escherichia coli* ST131 Clone. mBio.

[49] Lawley T.D., Gilmour M.W., Gunton J.E., Standeven L.J., Taylor D.E. (2002). Functional and mutational analysis of conjugative transfer region 1 (Tra1) from the IncHI1 plasmid R27. J. Bacteriol..

[50] Rooker M.M., Sherburne C., Lawley T.D., Taylor D.E. (1999). Characterization of the Tra2 region of the IncHI1 plasmid R27. Plasmid.

[51] Lawley T.D., Gilmour M.W., Gunton J.E., Tracz D.M., Taylor D.E. (2003). Functional and mutational analysis of conjugative transfer region 2 (Tra2) from the IncHI1 plasmid R27. J. Bacteriol..

[52] Gilmour M.W., Taylor D.E. (2004). A Subassembly of R27-encoded transfer proteins is dependent on TrhC nucleoside triphosphate-binding motifs for function but not formation. J. Bacteriol..

[53] Hüttener M., Prieto A., Aznar S., Bernabeu M., Glaría E., Valledor A.F., Paytubi S., Merino S., Tomás J., Juárez A. (2019). Expression of a novel class of bacterial Ig-like proteins is required for IncHI plasmid conjugation. PLoS Genet..

[54] Millan A.S., Heilbron K., MacLean R.C. (2014). Positive epistasis between co-infecting plasmids promotes plasmid survival in bacterial populations. ISME J..

[55] Pinto U.M., Pappas K.M., Winans S.C. (2012). The ABCs of plasmid replication and segregation. Nat. Rev. Microbiol..

