# Supplementary material for: Mechanisms of Transmission and Adaptation of tet(X4)-Positive IncHI1 Plasmids in XDR Escherichia coli from Pet Dogs: The Role of trhC, rsp, and the Tra1 Region
Source: Vet Sci. 2025 Apr 28;12(5):418. doi: 10.3390/vetsci12050418 (PMC12115866; doi:10.3390/vetsci12050418)
Supplement: Supplementary file 1 [file vetsci-12-00418-s001.zip › vetsci-3440071-supplementary.pdf]

Table S1 Primer sequence of this study

Primers	sequence (5′→3′)
pT28R-F1-F1	GATTCCGGGTACACAT
pT28R-F1-R1	CAGCGTTTGCACGATCC
pT28R-F1-F2	GGATCAGAACGTATAC
pT28R-F1-R2	TATACTCCCATGCCTC
pT28R-F2-F1	GCTGGAACGAGTACG
pT28R-F2-R2	CTTAACAGACTTGAC
pT28R-F2-F2	GCCAATAGACGAGCTG
pT28R-F2-R2	TATGGTCTATCTGCAC
pT28R-F3-F1	ATGAACCCATTCAAAGGCCG
pT28R-F3-R1	AGGACGGTGCAGCTCTGG
pT28R-F3-F2	CTGAGGGTAACAGGCGGT
pT28R-F3-R2	CACCAGATCCGTCACGAG
pT16R-F1-F1	CACGACATACCAAGT
pT16R-F1-R1	AACGAAGCGAGCGGGT
pT16R-F1-F2	GGGATCGCTCATATAG
pT16R-F1-R2	GACATGCGAAAGAGCT
tet(X4)-F	CTGATTCGTGTGACATCATCTTTTG
tet(X4)-R	GTTAAATTTCCCATTGGTCAGATTA
*fos*A3-F	GCGTCAAGCCTGGCATTT
*fos*A3-R	GCCGTCAGGGTCGAGAAA
floR-F	GTATGGGCACCTACTTCGTCT
floR-R	CAGCCCCAACGAAACCAGT
mcr-1-F	CGGTCAGTCCGTTTGTTC
mcr-1-R	CTTGGTCGGTCTGTAGGG

Table S2 Antimicrobial susceptibility testing of *E. coli* strains T28R, T16R and the transconjugants in this study

Strain	MICs (mg/L)													
	TGC	ERV	OMC	TET	DOX	AMP	CTX	IMP	COL	CIP	AMK	KAN	FFC	FOS
T16R	32	8	16	> 128	> 128	> 128	> 128	⩽0.125	8	32	128	128	128	256
T28R	32	8	16	> 128	128	> 128	> 128	⩽0.125	8	64	64	128	128	256
C600-pT16R-F1	2	1	0.5	32	32	32	32	⩽0.125	< 0.125	0.03	2	32	2	16
C600-pT28R-F1	4	2	2	128	64	> 128	32	⩽0.125	0.125	16	4	64	128	128
C600-pT28R-F2	4	4	2	128	64	> 128	64	⩽0.125	0.125	4	4	64	128	64
C600-pT28R-F3	4	2	2	128	128	> 128	32	⩽0.125	0.125	16	4	32	128	128

Note: TGC, tigecycline; ERV, eravacycline; OMC, omadacycline; TET, tetracycline; DOX, doxycycline; AMP, ampicillin; CTX, cefotaxime; COL, colistin; FFC, florfenicol; AMK, amikacin; KAN, kanamycin; CIP, ciprofloxacin; FOS, fosfomycin; IMP, imipenem.

Transconjugants C600-pT28R-F1, C600-pT28R-F2, and C600-pT28R-F3, carried fusion plasmids pT28R-F1, pT28R-F2, and pT28R-F3, respectively. The transconjugant C600-pT16R-F1 carried fusion plasmid pT16R-F1.

Table S3 Characterization of *E. coli s*trains T28R, T16R and their transconjugants used in this stay

strain	MLST	Plasmids	Plasmid type	Size (bp)	Self-transferability	Resistance gene	virulence genes
T28R	7366	Chromosome		4,606,706		*tet*(A)	*aslA*, *csgA*, *fdeC*, *fimH*, *gad*, *hlyE*, *nlpl*, *terC*, *yehA/B/C/D*
		pT28R -1	IncHI1	193,098	Conjugative	*tet*(X4), *lnu*(G), *aadA22*, *qnrS1*, *floR*, *bla*$_{\text{TEM-1}}$	none
		pT28R -2	IncF18:A–:B–	161,057	Conjugative	*aaa(3')-IVa*, *floR*, *sul2*, *bla*$_{\text{CTX-M-14}}$, *aph(3')-Ia*, *mph(A)*, *dfrA12*, *bla*$_{TEM-1}$, *fosA3, aadA2*	*anr*, *cma*, *cvaC*, *hlyF*, *iroN*, *iss*, *iucC*, *iutA*, *ompt*, *sitA*
		pT28R -3	IncI2	65,023	Conjugative	*mcr-1, bla*$_{\text{CTX-M-64}}$	none
C600-pT28R-F1		pT28R-F1	IncHI1 IncF18:A–:B–	354,983	Conjugative	*tet(X4)*, *lnu(G)*, *aadA22*, *qnrS1*, *floR*, *bla*$_{TEM-1}$, *aaa(3')-IVa*, *sul2*, *bla*$_{CTX-M-14}$, *aph(3')-Ia*, *mph(A)*, *dfrA12*, *fosA3, aadA2*	*anr*, *cma*, *cvaC*, *hlyF*, *iroN*, *iss*, *iucC*, *iutA*, *ompt*, *sitA*
C600-pT28R-F2		pT28R-F2	IncHI1 IncF18:A–:B–	354,983	Conjugative	*tet(X4)*, *lnu(G)*, *aadA22*, *qnrS1*, *floR*, *bla*$_{TEM-1}$, *aaa(3')-IVa*, *sul2*, *bla*$_{CTX-M-14}$, *aph(3')-Ia*, *mph(A)*, *dfrA12*, *fosA3, aadA2*	*anr*, *cma*, *cvaC*, *hlyF*, *iroN*, *iss*, *iucC*, *iutA*, *ompt*, *sitA*
C600-pT28R-F3		pT28R-F3	IncHI1 IncF18:A–:B–	263,549	Conjugative	*tet(X4)*, *lnu(G)*, *aadA22*, *qnrS1*, *floR*, *bla*$_{TEM-1}$, *aaa(3')-IVa*, *sul2*, *bla*$_{CTX-M-14}$,	*anr*, *cma*, *cvaC*, *hlyF*, *iroN*, *iss*, *iucC*, *iutA*, *ompt*, *sitA*

						aph(3')-Ia, *mph(A)*, *fosA3*	
T16R	48	Chromosome		4,639,361		*fosA3*, *bla*$_{TEM-1}$, *bla*$_{CTX-M-14}$, *bla*$_{TEM-214}$, *mdf(A)*, *bla*$_{TEM-1b}$	*anr*, *cib*, *cma*, *cvaC*, *hlyF*, *iroN*, *iss*, *ompT*, *sitA*
		pT16R -1	IncHI1	190,391	Conjugative	*tet(X4)*, *lnu(G)*, *aadA22*, *qnrS1*, *floR*, *bla*$_{TEM-1}$	none
		pT16R-2	IncF16:A–:B–	172,892	Conjugative	*dfrA17*, *AadA5*, *sul1*, *floR* *mph(A)*, aph(3')-Ia, *bla*$_{TEM-1}$, *sul2*, *aph(3'')-Ib*, *aph(6)-Id*, *tet(A)*	*asIA*, *csgA*, *fdeC*, *fimH*, *gad*, *hlyE*, *nlpl*, *terC*, *yehA*, *yehC*, *yehD*
		pT16R-3	IncX4	33,309	Conjugative	*mcr-1*	none
C600-pT16R-F1		pT16R-F1	none	108,982	Non-conjugative	*tet(X4)*, *bla*$_{TEM-1}$, *aph(3')-Ia*, *mph(A)*, *sul1*, *AadA5*, *dfrA17*	*cia*, *cma*, *cvaC*, *hlyF*, *iroN*, *iss*, *ompT*, *sitA*

Note: Transconjugants C600-pT28R-F1, C600-pT28R-F2, and C600-pT28R-F3, carried fusion plasmids pT28R-F1, pT28R-F2, and pT28R-F3, respectively. The transconjugant C600-pT16R-F1 carried fusion plasmid pT16R-F1. These transconjugants were screened on MacConkey agar supplemented with tigecycline (2 mg/L) and rifampicin (450 mg/L).

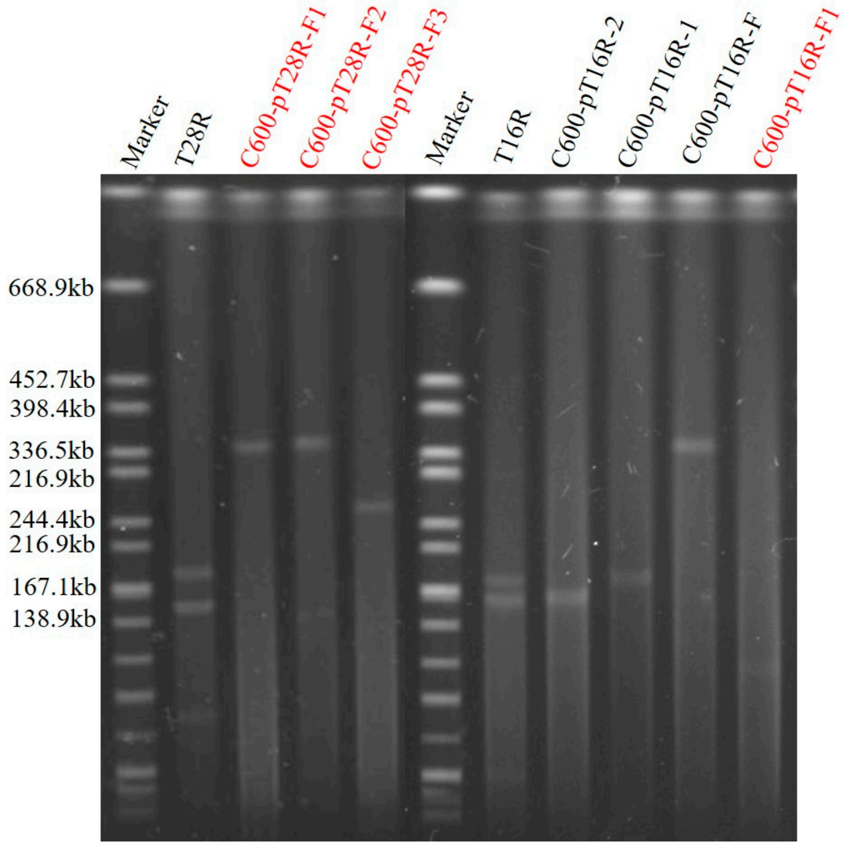

Fig. S1 S1-PFGE of *E. coli s*trains T28R, T16R and their transconjugants. Marker, *Salmonella* Braenderup H9812. S1-PFGE of *E. coli* strain T28R, T16R and transconjugants C600-pT28R-F1, C600-pT28R-F2, C600-pT28R-F3, C600-pT16R-F1, C600-pT16R-F, C600-pT16R-1, C600-pT16R-2.

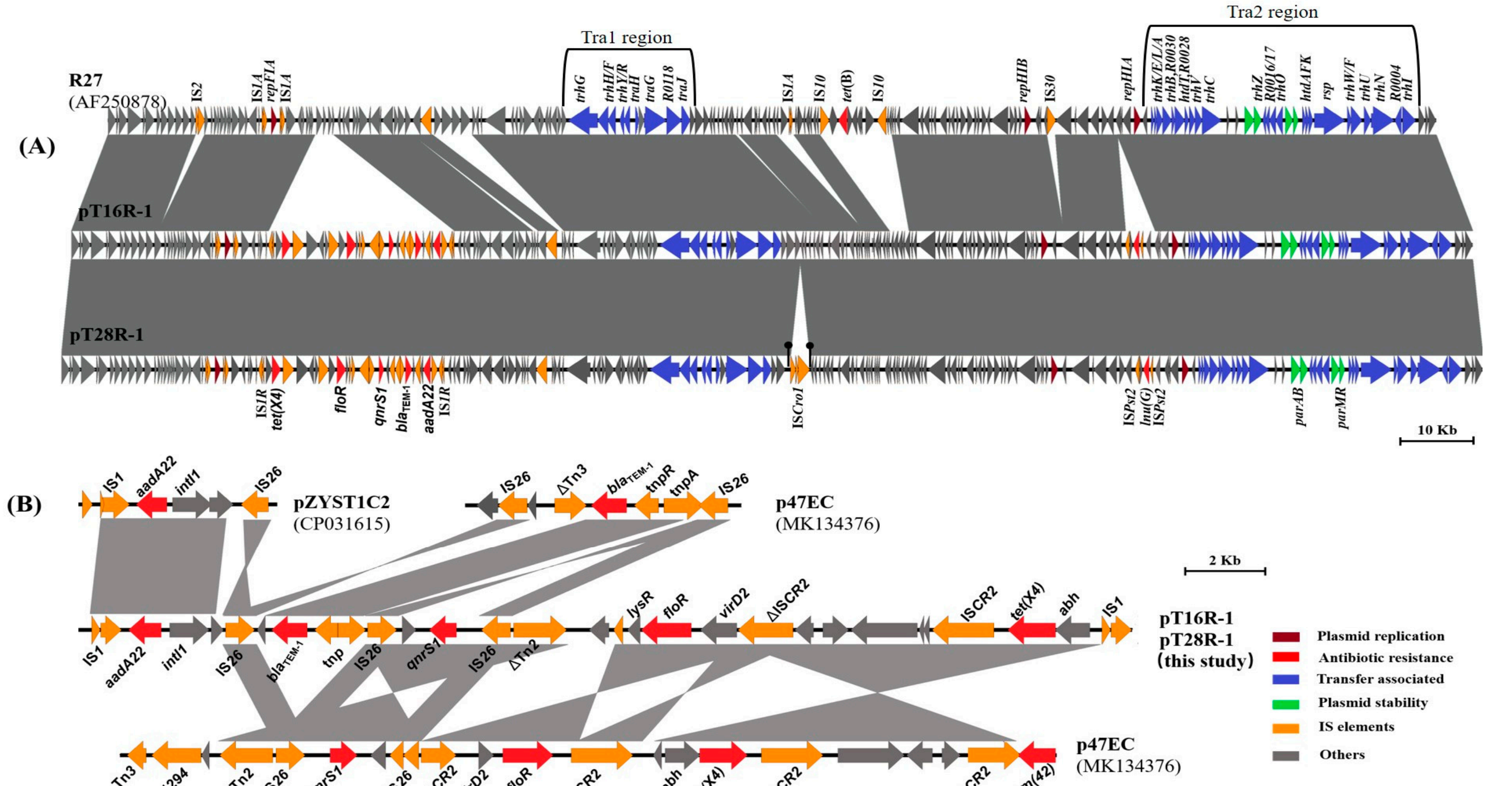

Fig. S2 Characteristics of the IncHI1 plasmid pT16R-1 and pT28R-1 carrying *tet*(X4). (a) The linear comparison of the plasmids pT16R-1 and pT28R-1 with the plasmid R27(AF250878). Filled circles on stalks are used to indicate TSDs (TAACCTTT). (b) Comparison of the MRR in pT16R-1 and pT28R-1 with that of other plasmids. Regions of >98% homology are marked by grey shading. Sequence comparisons and map generation were performed using BLAST and Easyfig (Version 2.1).

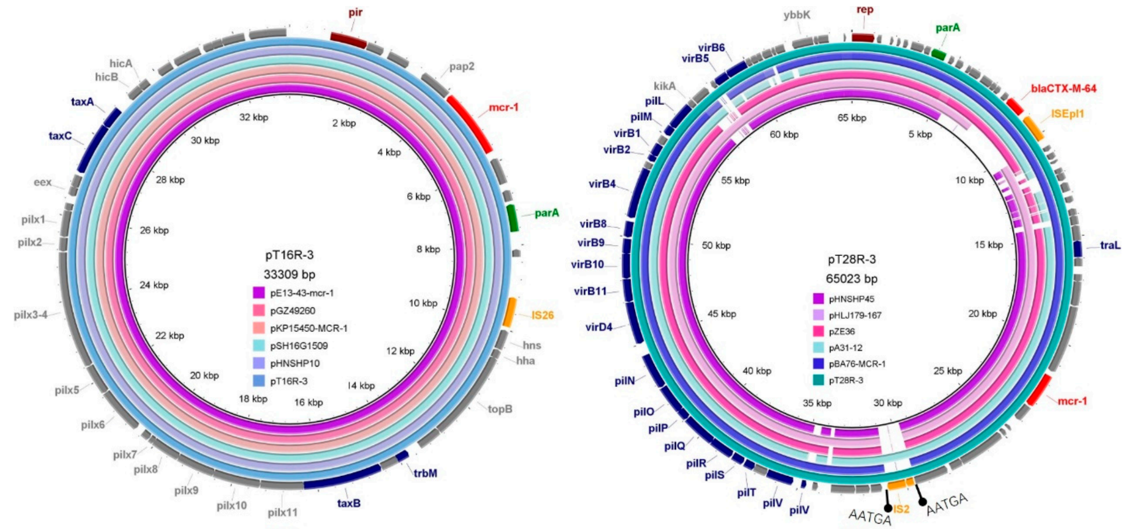

(a) (b)

Fig. S3 Structural comparisons of *mcr-1*-positive plasmids in this study and the similar plasmids in GenBank. (a) Comparison of pT16R-3 with similar plasmids. The outer ring represents the CDSs of the reference sequence pT16R-3. p92944-mph (MG838205) was in *E. coli* strain; pSH16G1509 (MK477615) and pKP15450-MCR-1 (MH715959) were respectively in *S. enterica* strain and *K. pneumoniae* strain; pGZ49260 (MG210937), pHNSHP10 (MF774182) and pE13-43-mcr-1 (MG747473) were in *E. coli* strains. **(b) Comparison of pT28R-3 with similar plasmids.** The outer ring represents the CDSs of the reference sequence pT28R-3. pHNSHP45 (KP347127), pHLJ179-167 (MN232211), pZE36 (KY802014), pA31-12 (KX034083), and pBA76-MCR-1 (KX013540) were all in *E. coli* strains of different origins. Filled circles on stalks are used to indicate TSDs (AATGA). Replicon genes are in maroon; transfer associated genes are in navy; resistance genes are in red; mobile elements are in orange; stability associated genes are in green; phage associated genes are in olive; heavy metal resistance genes are in black, and others are in gray. Sequence comparisons and map generation were performed using BLAST and BRIG.

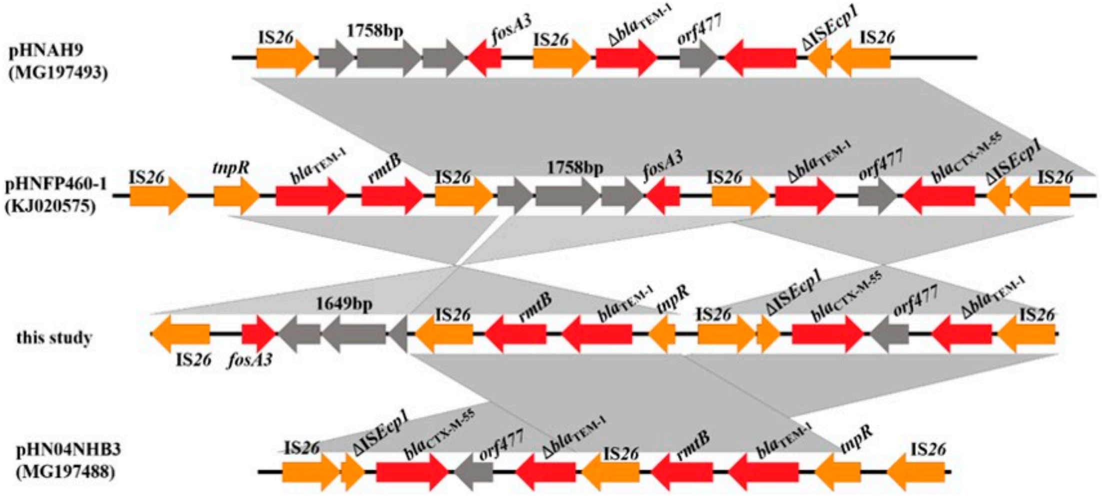

Fig. S4 Comparative analysis of the multidrug resistance regions. The arrows represent the transcriptional direction of the ORFs. Regions of 100% homology are shaded in grey. Resistance genes are in red; mobile elements are in orange; and others are in black gray. Sequence comparisons and map generation were performed using BLAST and Easyfig (Version 2.1).

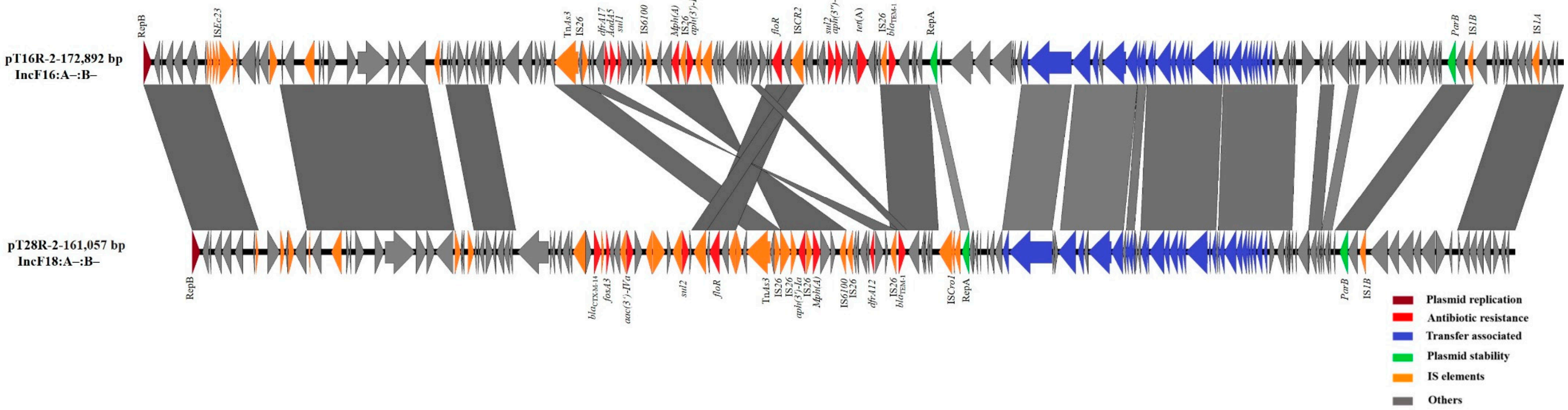

Fig. S5 Comparative analysis of plasmids pT28R-2 and pT16R-2. The arrows represent the transcriptional direction of the ORFs. Regions of 100% homology are shaded in grey. Resistance genes are in red; mobile elements are in orange; and others are in black gray. Sequence comparisons and map generation were performed using BLAST and Easyfig (Version 2.1).
